# Triggering of viral RNA sensors induces CD55 expression on synovial fibroblasts

**DOI:** 10.1186/1479-5876-9-S2-P41

**Published:** 2011-11-23

**Authors:** Olga N Karpus, Kirstin M Heutinck, Paul JM Wijnker, Paul Peter Tak, Jorg Hamann

**Affiliations:** 1 Dept. of Experimental Immunology, Academic Medical Center, Amsterdam, The Netherlands; 2 Renal Transplant Unit, Academic Medical Center, Amsterdam, The Netherlands; 3 Division of Clinical Immunology and Rheumatology, Academic Medical Center, Amsterdam, The Netherlands

## Background

CD55 (decay-accelerating factor) is a complement-regulatory protein, which is highly expressed by fibroblast-like synoviocytes (FLS). CD55 is also a ligand for CD97, an adhesion-type G protein-coupled receptor abundantly present on leukocytes. We recently showed that lack of either CD55 or CD97 ameliorates disease in murine collagen-induced and K/BxN serum transfer models of arthritis (Arthritis Rheum. 2010 Apr;62(4):1036-42). Little is known regarding the regulation of CD55 expression in FLS. We therefore investigated the effect of Toll-like receptors ligation and pro-inflammatory cytokines on CD55 expression.

## Materials and methods

Synovial fibroblasts, obtained from biopsy samples of arthritis patients, were cultured and stimulated with cytokines (TNF, IFNγ, IL-1β, IL-6, IFNα) or TLR ligands (LTA, poly(I:C), LPS, imiquimod, CpG). Expression of CD55 was measured by flow cytometry using domain-specific monoclonal antibodies and recombinant CD97-loaded fluorescent beads. Chloroquine was used to inhibit TLR3 activity. Upregulation and functionality of dsRNA sensors in response to poly(I:C) or 5’-triphosphate RNA was analyzed by PCR. Apoptosis was measured by PI/annexinV staining and was blocked with the pan-caspase inhibitor Q-VD-OPH.

## Results

Cultured synovial fibroblasts of patients with rheumatoid arthritis (RA), osteoarthritis, psoriatic arthritis, and spondylarthritis express equal amount of CD55. Stimulation of RA-FLS with IL-1β (p=0.02) and poly(I:C) (p=0.001) induced a significant upregulation of CD55. Engagement of TLR3 by the dsRNA analog poly(I:C) was confirmed using chloroquine, an inhibitor of endosomal acidification that impairs TLR3 signaling. Synovial fibroblasts also expressed the cytoplasmic dsRNA sensors melanoma differentiation-associated gene 5 (MDA5) and retinoic acid-inducible gene I (RIG-I). Stimulation of these receptors with either poly(I:C) or 5'-triphosphate RNA induced CD55 expression, but, in case of MDA5, also induced significant cell death (p<0.001) that was caspase-dependent. Upregulation of CD55 in response to dsRNA receptor activation increased the binding capacity of synovial fibroblasts for CD97-loaded beads.

## Conclusions

We identify dsRNA as a potent inducer of CD55 upregulation on synovial fibroblasts. Our findings suggest that CD55 induction by viral dsRNA or dsRNA may facilitate the accumulation of CD97-expressing inflammatory immune cells in the synovial tissue.

